# Use of Integrated Optical Clearing and 2-Photon Imaging to Investigate Sex Differences in Neuroimmune Interactions After Peripheral Nerve Injury

**DOI:** 10.3389/fcell.2021.624201

**Published:** 2021-02-18

**Authors:** Thomas A. Szabo-Pardi, Umar M. Syed, Zachary W. Castillo, Michael D. Burton

**Affiliations:** Neuroimmunology and Behavior Laboratory, Department of Neuroscience, Center for Advanced Pain Studies (CAPS), School of Behavioral and Brain Sciences, University of Texas at Dallas, Richardson, TX, United States

**Keywords:** optical clearing, nerve injury, sex differences, neuroimmune interactions, 2-photon, macrophage, DRG

## Abstract

Peripheral nerve injury induces a myriad of immune-derived symptoms that negatively impacts pain, depression, and overall quality of life. Neuroimmune differences underlie sexual dimorphisms in various pain states. The innate immune system is a source of these sex differences, which promotes inflammation and pro-nociception through bidirectional signaling with the nervous system. Spatiotemporal interactions between leukocytes and sensory neurons could hold the key to explain ascribed differences between sexes. To date, studies have found it difficult to display these interactions. We are poised to answer important questions regarding the recruitment of peripheral leukocytes to key tissues of the pain system, the dorsal root ganglia (DRG) and sciatic nerve after nerve injury. We optically clear whole DRGs and sciatic nerves and concomitantly use multi-photon microscopy and transgenic reporter lines, to visualize leukocyte dynamics involved in neuropathic pain development following nerve injury. We observed robust sexual dimorphisms in leukocyte recruitment to the lumbar DRGs after nerve injury. We also assessed immune cell size and morphology to understand activation states in the context of nervous tissue inflammation. The altered mechanisms by which the male and female immune systems respond to nerve injury are still topics of further research, however; the continued use of next-generation imaging with advanced whole tissue image analysis remains an important tool in understanding the reciprocal interactions between neuronal and non-neuronal cells.

## Introduction

Peripheral nerve injury often results in neuropathic pain, which is defined by trauma or lesions that disrupt the somatosensory systems. Injury-induced neuropathic pain is estimated to occur in over 30% of patients following routine operations (Kehlet et al., 2006). Patients often report higher levels of comorbidities, such as depression and sleep disorders which further contribute to increased pathological clinical outcomes and significantly reduce quality of life (McDermott et al., 2006; Cohen et al., 2015). Although the prevalence of chronic pain continues to rise, the number and effectiveness of existing therapeutics remains limited (Finnerup, 2015). The increasing incidence of neuropathic pain has piqued interest in understanding the key immunologic processes involved. Previous studies have found a clinically observed difference in the prevalence and perception of pain in males vs. females (Fillingim et al., 2009; Mogil, 2012). However, there is still a dearth of knowledge on the sexual dimorphisms observed in leukocyte trafficking, morphology, and neuroimmune interaction.

Macrophages, key myeloid-derived leukocytes have been shown to play a key role in facilitating maladaptive nociception following nerve injury (Zhuo et al., 2011). Moreover, the peripheral immune system, specifically macrophages, play a pivotal role in sensitization of sensory neurons (Lindborg et al., 2018). Macrophages in the dorsal root ganglia (DRG) and sciatic nerve (ScN) play a critical role in both the initiation and maintenance of neuropathic pain by enhancing sensory neuron transduction and excitability (Basbaum et al., 2009). We hypothesize that using intact tissue for 3D rendering and morphologic analysis; we will be able to differentiate spatial resolution of macrophage infiltration and their interactions with sensory neurons in a sex-specific fashion. Recent studies have shown that these macrophages demonstrate robust molecular crosstalk with sensory neurons (Yu et al., 2020). This remains an integral process of not only pain induction as a protective mechanism, but also a transition to maladaptive chronic pain in some instances (Renthal et al., 2020). Discovering the sex-dependent roles of macrophages in tissue injury is paramount as the incidence macrophage-dependent chronic pain in females is lower than in males (Wiesenfeld-Hallin, 2005; Agalave et al., 2020; Rudjito et al., 2020). As such, identifying sex differences in macrophage biology will serve as a foundation for future studies aimed at exploiting immunological regulation in pain and will serve to create a more tailored approach to therapeutics.

Our group has recently developed a technique for intravital imaging using transgenic reporter mice and multiphoton microscopy (Szabo-Pardi et al., 2019). We wanted to adapt these methods to conceptualize the immune response to injury. Changes in cellular morphology has been associated with changes in functionality of cells (McWhorter et al., 2013). We used transgenic reporter animals, fluorescently labeled (ROSA26^tdTomatoLSL^) × LysozymeM:cre (LysM^+^)-expressing leukocytes (LysM^tdT+^). LysM is an antimicrobial enzyme (encoded by the *Lyz2* gene) that breaks down gram-positive bacterial cell walls and is predominately expressed by circulating neutrophils and tissue macrophages (Goren et al., 2009). Tissue macrophages have been shown to be upregulated within days after peripheral injury and are known to play a role in injury-induced sensitization of sensory neurons (Clausen et al., 1999; Rittner, 2005; Kiguchi et al., 2018). Prior studies have used techniques, such as flow cytometric analysis to quantify recruitment or infiltration of macrophages into peripheral nervous tissue, however, there are inherent limitations using this method (Ghasemlou et al., 2015; Lopes et al., 2017). Notably, a lack of clarity regarding pathogenesis and spatiotemporal visualization of macrophages in the extracellular space. Moreover, studies that investigate recruitment of any immune cell to peripheral nervous tissues rarely use both sexes, making it difficult to draw apt comparisons (Kwon et al., 2013; Schmid et al., 2013).

To provide a more robust understanding of interactions in nervous tissue after injury, we cleared tissue (Sca*l*eS1) to enable visualization of tdTomato-tagged macrophages via multiphoton microscopy. Not only does this technique preserve the integrity of extracted tissues, but it also provides an accurate representation of cell dynamics in a diseased state (Gómez-Gaviro et al., 2020). The ability to use whole, unsectioned tissue provides a clear advantage over conventional methods, which is made possible by visualizing the spatial relation of neuronal and non-neuronal immune cells in a three-dimensional (3-D) model. In this study, we find that male mice exhibit more robust infiltration of LysM^tdT+^ macrophages after injury as compared to females. Additionally, we took advantage of advanced image analysis in concert with our experimental approach to group and classify the morphology of these macrophages. Recent literature has shown that macrophages exemplify distinct morphological changes differentiating into M1 and M2 phenotypes, pro-inflammatory or anti-inflammatory functions, respectively (McWhorter et al., 2013). In response to physiological changes and cytokine signaling, M2 macrophages are associated with an elongated, prolate morphology while M1 macrophages are associated with an oblate, flattened morphology (Bertani et al., 2017). This serves as an indispensable tool in our approach to elucidate sex differences after nerve injury and can be adapted to address the gap in understanding the intimate interactions between leukocytes and neurons in other aspects in neuroimmunology research.

## Materials and Methods

### Laboratory Animals

All animal experiments were carried out in accordance with protocols approved by the Institutional Animal Care and Use Committee of the University of Texas at Dallas. Mice were housed (4–5 per cage) in a temperature-controlled facility (20–25°C) and maintained on a 12-h light/dark cycle (lights on: 6 a.m./lights off: 6 p.m.). Mice had *ad-libitum* access to food and water and were 8–12-weeks-old during the experiments (male, 25–30 g; female, 20–25 g). Transgenic mice expressing NLS-Cre recombinase under control of the endogenous *Lyz2* promoter/enhancer elements (LysM) were obtained commercially from Jackson (Stock no: 004781). Characterization of these mice showed that heterozygous cre animals have no pain phenotype and normal electrophysiological properties (Clausen et al., 1999). Furthermore, transgenic mice expressing a loxP-flanked *STOP* cassette preventing expressing of tdTomato (red fluorescent protein) were purchased from Jackson (Stock no: 007909) and bred with LysM^cre+^ animals in-house (LysM^cre+^ × ROSA26^LSLtdTomato^ = LysM^tdT^) and used for all behavioral and biochemical assays. All animals used were heterozygous for LysM^cre^ and had at least one copy of tdTomato. All strains were backcrossed to maintain C57BL/6J genetic background with animals from Jackson Lab (stock no. 000664).

### Surgical Procedures

The spared nerve injury (SNI) model of neuropathic pain was used. Baseline values for behavioral experiments were established 24-h prior to surgery. Mice were anesthetized under isoflurane anesthesia (1.0–2.5%). The ipsilateral thigh was shaved and cleaned with betadine (Dynarex, NY, USA; cat no. 1425) and 70% ethanol (Decon Labs, PA, USA; cat no. 2701). The skin and muscle of the ipsilateral thigh were incised with a #11 scalpel (Thermo Fisher, MA, USA; cat no. 22-079-691) and the sciatic nerve along its three branches (common peroneal, tibial, and sural) were exposed. A tight ligature using a 5-0 silk suture (VWR, PA, USA; cat no. MV-682) was placed around the proximal tibial and common peroneal branches, after which the nerves distal to the ligature were transected, taking care to not stretch or damage the sural nerve. The skin was closed using an auto clip (Fine Science Tools, CA, USA; cat no. 12022-09) and mice were returned to their home cages to recover (Decosterd and Woolf, 2000). Sham surgeries were done identically to the SNI surgery; however, no portion of the sciatic nerve was ligated or transected. Following surgery, mice are subcutaneously administered a single dose of Gentamicin (5 mg/mL) (Sigma-Aldrich, CA, USA; cat no. G1272) as a prophylactic antibiotic. All mice were then returned to their home cages for recovery and monitored daily.

### Behavioral Testing

To measure mechanical hypersensitivity, mice were individually placed on an elevated wire grid inside acrylic behavior racks and allowed to habituate for ~2-h. Behavior racks were cleaned with a 1:3 ratio of a natural all-purpose and deodorant-free cleaner (Seventh Generation™, VT, USA; cat no. 22719BK-5) and DI water and wiped dry to eliminate odor cues between each reading, baseline, and experiment. The ipsilateral hind paw was then stimulated with von Frey filaments (Stoelting Co., IL, USA; cat no. 58011) using the up-down experimental paradigm (Chaplan et al., 1994). To assess cold allodynia (cold response) in our SNI model, mice were individually placed on the same elevated wire grid and behavior racks and allowed to habituate for ~2-h before testing. Approximately 100 μL of biological grade acetone (Fisher Scientific, MA, USA; cat no. AI6P-4) was then applied to the lateral aspect of the ipsilateral hind paw using a 1 mL syringe (VWR, PA, USA; cat no. 309659) attached to a blunted 25 G needle (VWR, PA, USA; cat no. 305125). Cold response was assessed by paw licking, shaking, grooming behaviors and were measured over a 60-s period (Yoon et al., 1994). Baseline values were taken 24-h prior to performing surgery. Mechanical hypersensitivity and cold allodynia were then measured on post-operative days 1, 3, and 5. Mechanical measures were always taken before cold response. All behavioral testing was done between 10 a.m. and 2:00 p.m. Experimenters were blinded to genotype, surgery, or both.

### Optical Clearing

Five days post-SNI, mice were deeply anesthetized using a mixture of ketamine (80 mg/kg) and xylazine (12 mg/kg), injected intraperitoneally, and were transcardially perfused with 10 mL of ice cold 1 × PBS (Thermofisher, MA, USA; cat no. BP3994) and then 10 mL of ice cold 4% paraformaldehyde (Sigma-Aldrich, CA, USA; cat no. F8775) using a 25G winged infusion set (Thermofisher, MA, USA; cat no. 14-840-37). Lumbar dorsal root ganglia (DRGs) (L4-5) and sciatic nerves (ScNs) were collected in 2 mL microcentrifuge tubes (Eppendorf, CT, USA; cat no. 022-43-104-8) and post-fixed in 1.5 mL of 4% paraformaldehyde (Sigma-Aldrich, CA, USA; cat no. F8775) (made in 1 × PBS) for 4-h. Fixed tissues were then transferred to a 2 mL microcentrifuge tube containing 1.5 mL of 20% sucrose solution (VWR, PA, USA; cat no. 0335-1KG) (made in 1 × PBS) for 48 h. Following cryoprotection, tissues were then transferred to a 5 mL microcentrifuge tube (Eppendorf, CT, USA; cat no. 0030-119-401), immersed in 1.5–4 mL of Sca*l*eS1 solution and placed on a tissue nutator for ~10–14 days to achieve optimal tissue clarity (Hama et al., 2015). This was to enhance perfusion of the extracted tissues. Sca*l*eS1 solution was aspirated and replaced every 48-h with fresh solution. The following reagents were used to prepare Sca*l*eS1 solution: 4 M urea crystals (Thermofischer, MA, USA; cat no. 29700), 0.1% (wt/vol) Triton X-100 (Sigma-Aldrich, CA, USA; cat no. X100), and 10% (wt/wt) glycerol (Fischer Scientific, MA, USA; cat no. BP229-1). In brief, urea crystals were dissolved in water using a stir bar Thermofisher, MA, USA; cat no. F37180). Next, the triton x-100 was added along with glycerol and was left to mix for an hour. The solution was made and allowed a minimum of 48-h to equilibrate before use and stored at room temperature.

### Multiphoton Microscopy

Optically cleared DRGs and ScNs were embedded in single 13 mm glass-bottomed cell culture plates (Thermofisher, MA, USA; cat no. 150680) using 0.5% (w/v) agarose (VWR, PA, USA; cat no. MPN605) (dissolved in ultra-pure ddH_2_O) as an immobilization medium. Upon polymerization of the agarose, ~1 mL of Sca*l*eS1 solution was pipetted into the culture plates to ensure coverage and adequate hydration of immobilized samples. Samples were individually imaged using an Olympus MPE-RS TWIN multiphoton microscope outfitted with dual excitation lasers (Spectra Physics INSIGHT DS+ -OL pulsed IR LASER, tunable from 680 to 1,300 nm, 120 fs pulse width at specimen plane and SPECTRA PHYSICS MAI TAI HP DEEP SEE-OL pulsed IR LASER, tunable from 690 to 1,040 nm, 100 fs pulse width at specimen plane). We have established optimal parameters for multiphoton microscopy in a previous study (Szabo-Pardi et al., 2019). In brief, using these excitation lasers in combination with a XLPLN25XWMP2 Olympus ultra 25 × MPE water-immersion objective (1.05 NA, 2 mm WD) we were able to image tdTomato-positive LysM^+^ cells (LysM^tdT+^) (1,100 nm). Tissues were scanned using a galvanometer scanning unit at 10 us/pixel; 1:1 aspect ratio; 0.5 step size; 512 × 512 area and images were acquired with 2-channel multi-alkali photomultiplier tubes (PMTs). Z-stack images were acquired of the entire sample (Y-plane) of the DRG and distal/proximal portions of the ScN. A step size of 1 um per slice was used and images were between 300 and 400 slices. FVMPE-RS system software (FluoView) was used to acquire images. Raw Z-stack images were then exported to Imaris imaging software for appropriate processing and analysis. Images were acquired by a blinded experimenter.

### Image Analysis

Analysis of ScN and DRG tissues LysM^tdT+^ labeled macrophages was done with Imaris Software (Oxford Instruments, version 9.0.1). Previously acquired z-stacks are put into Imaris where each pixel from the 2D section was converted into a 3D voxel. This information was then used to reconstruct the original 3D object that spanned across the z-stacks. Images were imported into Imaris's Arena and viewed within the 3-D view of the Surpass. The Surfaces visualization is a computer-generated representation of the specified gray value range in the data set. In order to visualize the range of interest of an object's volume, an artificial solid object is created from which measurements can be derived. Surfaces were created using background subtraction for td-Tomato positive cells in the Z-stack images. A filter based on number of voxels was used to remove both artifacts and large neuronal cells within the image. Using the most representative image for DRGs and ScNs, creation parameters were made using the corresponding creation wizard in Imaris's Surfaces feature. Surfaces of the LysM^tdT+^ macrophages were then measured for cell count, ellipticity, volume, and sphericity. In order to normalize cell count via volume of tissue, surfaces were created for the whole DRG and sections of ScN using absolute intensity thresholding. Both proximal and distal sections of ScN were analyzed and data points were labeled accordingly to determine spatial differences in macrophage infiltration and activation along the ScN. All analyses were performed by an experimenter blinded to sex, genotype, treatment, and tissue type.

1. Sphericity—Given as a value from 0.01 to 1.00 with 1.00 being a perfect sphere in which the x, y, and z axes are all equal length. The sphericity of a particle is the ratio of the surface area of an equal-volume sphere to the actual surface area of the particle. The closer to 0 this value is the less spherical and more ellipsoid the shape is.

$$\begin{array}{l} \psi =\frac{\pi^{1/3}{\left(6V_{p}\right)}^{2/3}}{A_{p}} \end{array}$$

v_p_ = volume of the particle

A_p_ = surface area of the particle

2. Prolate ellipticity—Given as a value from 0.01 to 1.00 with 1.00 representing an ellipsoid with one axis significantly longer than the others. A prolate ellipticity value moving toward 0 represents the lengths of the x, y, and z axes becoming more even. Values closer to 0 represent a more spherical shape while values closer to 1 represent a more elongated shape. A more elongated shape is typically associated with an M2 phenotype.

$$\begin{array}{l} e_{prolate}=\frac{2a^{2}}{a^{2}+b^{2}}▪\left(1-\frac{a^{2}+b^{2}}{2c^{2}}\right) \end{array}$$

3. Oblate ellipticity—Given as a value from 0.01 to 1.00 with 1.00 representing an ellipsoid with two axes equal in length but longer than the third. An oblate ellipticity value moving toward 0 represents the lengths of the x, y, and z axes becoming more even. Values closer to 0 represent a more spherical shape while values closer to 1 represent a more flattened shape. A more flattened shape is typically associated with an M1 phenotype.

$$\begin{array}{l} e_{oblate}=\frac{2b^{2}}{b^{2}+c^{2}}▪\left(1-\frac{2a^{2}}{b^{2}+c^{2}}\right) \end{array}$$

### Statistical Analysis

Prism 8.01 software (GraphPad, San Diego, CA, USA) was utilized to generate all graphs and statistical analysis. Single comparisons were performed using Student's *t*-test, and multiple comparisons were performed using a one-way or two-way ANOVA with Bonferroni *post-hoc* tests for across-group comparisons. All data are represented as the standard error of the mean (SEM). A *p*-value of <0.05 was used to determine statistical significance. Blinded experimenters performed all experiments and analysis.

## Results

### Use of Interdisciplinary Techniques to Investigate the Macrophage Response During Peripheral Nerve Injury

Macrophages are implicated in the development of pain following nerve injury and have complex immunologic, neurologic, and physical facets (Raoof et al., 2018). To improve our understanding of the dynamic immune response after peripheral nerve injury, we designed an interdisciplinary approach combining advanced multiphoton microscopy, Sca*l*eS1 tissue clearing and a well-established nerve injury model (SNI) Using these techniques, we were able to address some of the limitations of previous studies investigating the macrophage response to nerve injury. Primarily, these limitations include: a lack of appropriate male and female representation in data sets, an inability to assess morphological changes in macrophages while preserving the integrity of the microenvironment, and skewed information resulting from single-slice imaging analysis as opposed to whole tissue. While these studies greatly improve our understanding on the dynamic nature of macrophage recruitment and activation, they highlight a need to develop integrative techniques to improve our approach.

### Male and Female Mice Exhibit Robust Pain Behaviors Following SNI

**Figure 1:**
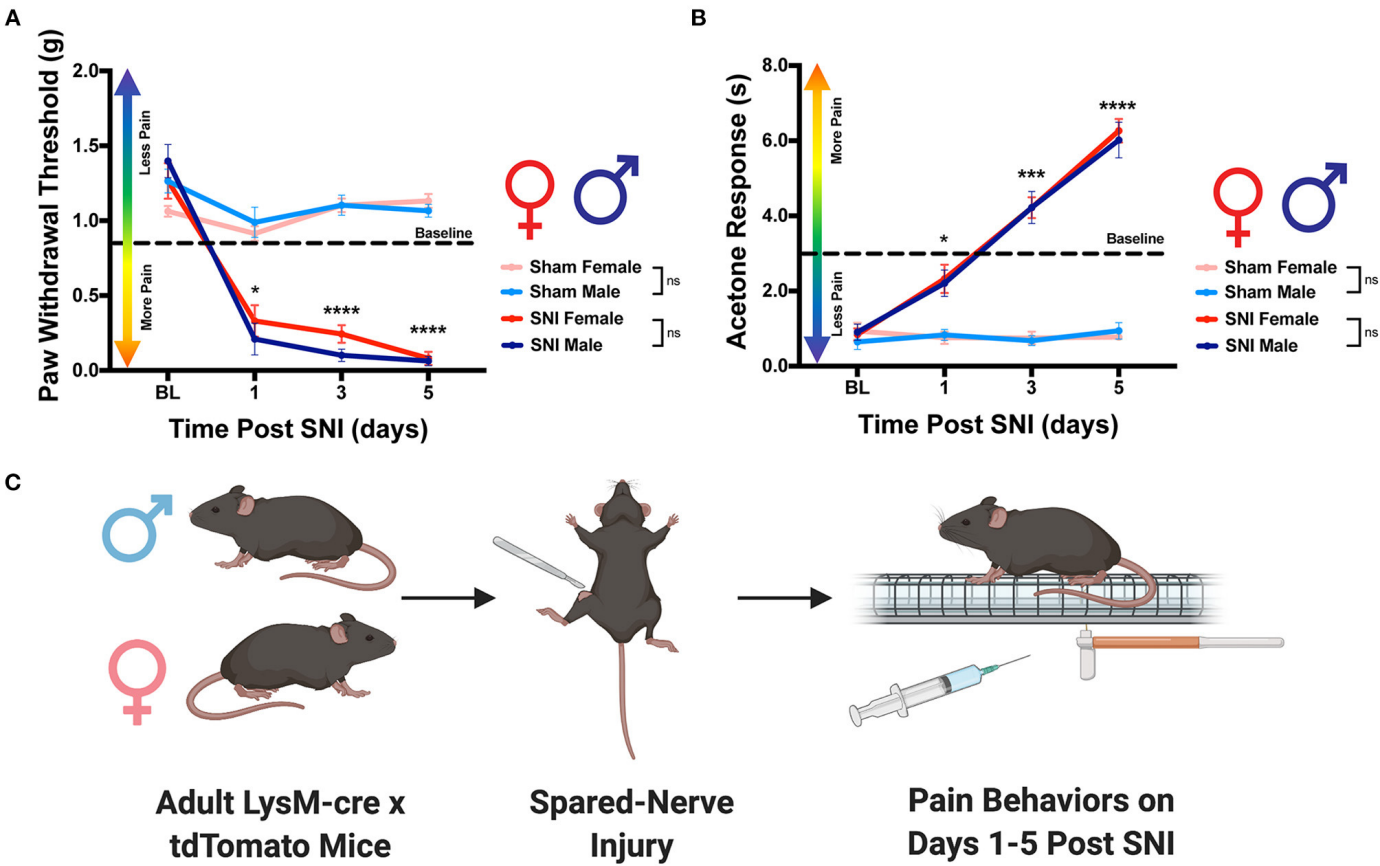
Development of neuropathic pain behaviors following SNI. Male and female LysM^tdT+^ mice were tested for mechanical hypersensitivity and cold allodynia on days 1, 3, and 5 post-SNI. **(A)** Male (*n* = 7) and Female (*n* = 7) LysM^tdT+^ mice exhibit significantly reduced mechanical withdrawal thresholds post-SNI as compared to their respective sham controls (*n* = 7). **(B)** Male (*n* = 7) and Female (*n* =7) LysM^tdT+^ mice exhibit significantly elevated response latency to acetone application as compared to their respective sham controls (*n* = 7). **(C)** Graphical representation of the experimental timeline where male (*n* = 7) and Female (*n* = 7) LysM^tdT+^ mice were given SNI and were assessed for mechanical hypersensitivity and cold allodynia on days 1, 3, and 5 post-SNI. ^*^p < 0.05; ^***^p < 0.001; ^****^p < 0.0001. BL, Baseline. Made using BioRender.com.

To adequately assess the macrophage response to nerve injury, spared nerve injury (SNI) was performed to induce a pain state in both sexes. We chose this specific model of neuropathic pain because it has been shown to cause prolonged changes in behavioral phenotypes as well as immune cell activation (Raoof et al., 2018). Moreover, the etiology of neuropathic pain that develops after SNI closely mimics the cardinal symptoms of clinically described neuropathic pain (Chen et al., 2015). In order to confirm that our procedure induced a pain state, we assessed mechanical hypersensitivity and cold allodynia in mice that received either SNI or sham. As expected, we found that males that received SNI exhibited significantly reduced paw withdrawal thresholds on days 1, 3 and 5 as compared to their sham counterparts. We report similar results in females where mice that received SNI had significantly reduced paw withdrawal thresholds on day 1, 3, and 5 as compared to sham controls (Figures 1A,C). Moreover, male mice that received SNI exhibited an elevated behavioral response to application of acetone to the ipsilateral hind paw on days 3 and 5 as compared to their sham counterparts. Similarly, female mice that received SNI exhibited an elevated behavioral response to application of acetone to the ipsilateral hind paw on days 1, 3, and 5 as compared to sham controls (Figures 1B,C). Lastly, we find no significant sex differences in the onset of mechanical hypersensitivity or cold allodynia after SNI. Taken together, these data indicate SNI induced robust pain behaviors before, and up to the day mice were euthanized and tissues were collected for analysis.

### SNI Induces Macrophage Recruitment, Activation, and Morphological Changes in the DRG

**Figure 2:**
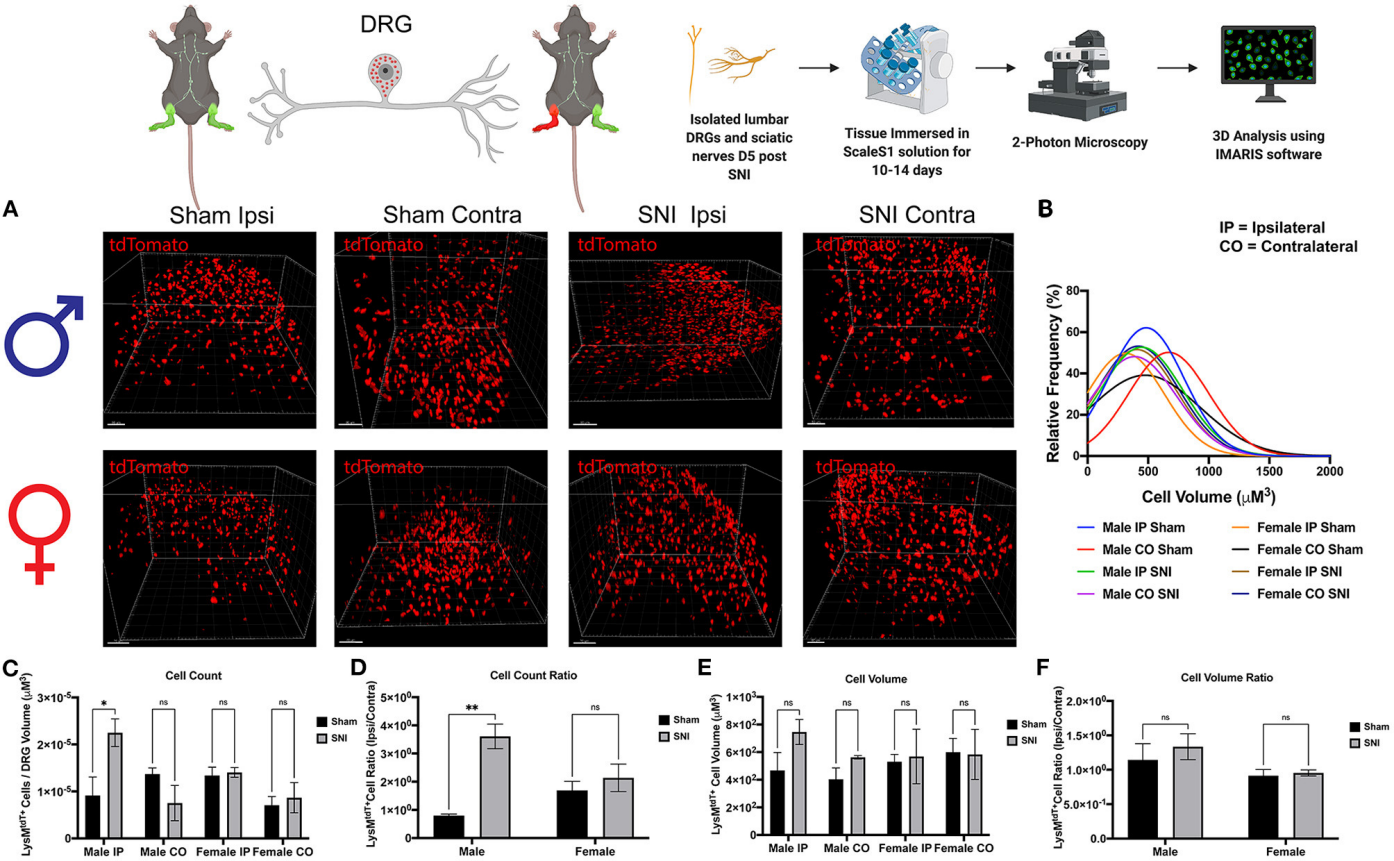
Infiltration of LysM^tdT+^ macrophages in the lumbar DRGs 5 days post-SNI. Male and female LysM^tdT+^ mice had DRGs harvested 5 days post-SNI. Tissues were processed and cleared using ScaleS1 solution for 10–14 days and imaged using 2-photon microscopy. **(A)** Representative images from male and female LysM^tdT+^ (red; 1,100 nm) DRGs (*n* = 3). **(B)** Frequency distribution histogram comparing the frequencies of LysM^tdT+^ cell volumes in control and injured male and female lumbar DRGs. **(C)** Quantification of LysM^tdT+^ macrophage infiltration in the lumbar DRGs 5 days post-SNI (*n* = 3) in males and females. **(D)** Quantification of LysM^tdT+^ macrophage infiltration in the lumbar DRGs 5 days post-SNI as a ratio of ipsilateral over contralateral DRG (*n* = 3) in males and females. **(E)** Quantification of LysM^tdT+^ macrophage volume in the lumbar DRGs 5 days post-SNI (*n* = 3) in males and females. **(F)** Quantification of LysM^tdT+^ macrophage cell volumes in the lumbar DRGs 5 days post-SNI as a ratio of ipsilateral over contralateral DRG (*n* = 3) in males and females. ^*^p < 0.05; ^**^p < 0.01. Scale Bar: 100 μm. IP, Ipsilateral; CO, Contralateral. Made using BioRender.com.

To explore our initial hypothesis regarding sex differences in macrophage infiltration, lumbar DRGs (L4-5) were harvested from male and female LysM^tdT+^ reporter mice 5 days post-SNI. We integrated Sca*l*eS1 whole tissue clearing with multiphoton imaging in both male and female DRGs to visualize macrophage recruitment, activation, and changes in morphology (Figure 2A). Imaris image analyses revealed significant differences in macrophage recruitment to the DRG following SNI. We found that injured (SNI) male ipsilateral DRGs displayed significantly increased amounts of LysM^tdT+^ macrophages compared to the contralateral (uninjured) DRGs. Moreover, SNI induced significantly elevated LysM^tdT+^ macrophage recruitment to the ipsilateral DRG as compared to the sham surgery. Surprisingly, we do not report a significant increase in LysM^tdT+^ macrophage recruitment to the ipsilateral DRG in female mice after SNI. This remains true in the sham groups as well (Figure 2C). To account for differences in resident and infiltrating macrophages, we assessed the ratio of LysM^tdT+^ macrophages in the ipsilateral (injured) DRG with the contralateral (uninjured) DRG. Again, we found that males display significantly upregulated LysM^tdT+^ macrophages to the ipsilateral DRG. This is indicative of an upregulation of infiltrating macrophages (Figure 2D). To better understand the activation states of these infiltration macrophages, we analyzed the distribution and relative frequencies of LysM^tdT+^ macrophage volumes after SNI. We report no significant findings; however, male mice exhibit a trend of larger cell volumes in the injured ipsilateral DRG (Figures 2B,E,F). While these findings are not statistically significant, considered with the robust recruitment of LysM^tdT+^ macrophages, we can conclude that there is a biologically relevant upregulation and activation of macrophages in the DRG following SNI in male mice.

**Figure 3:**
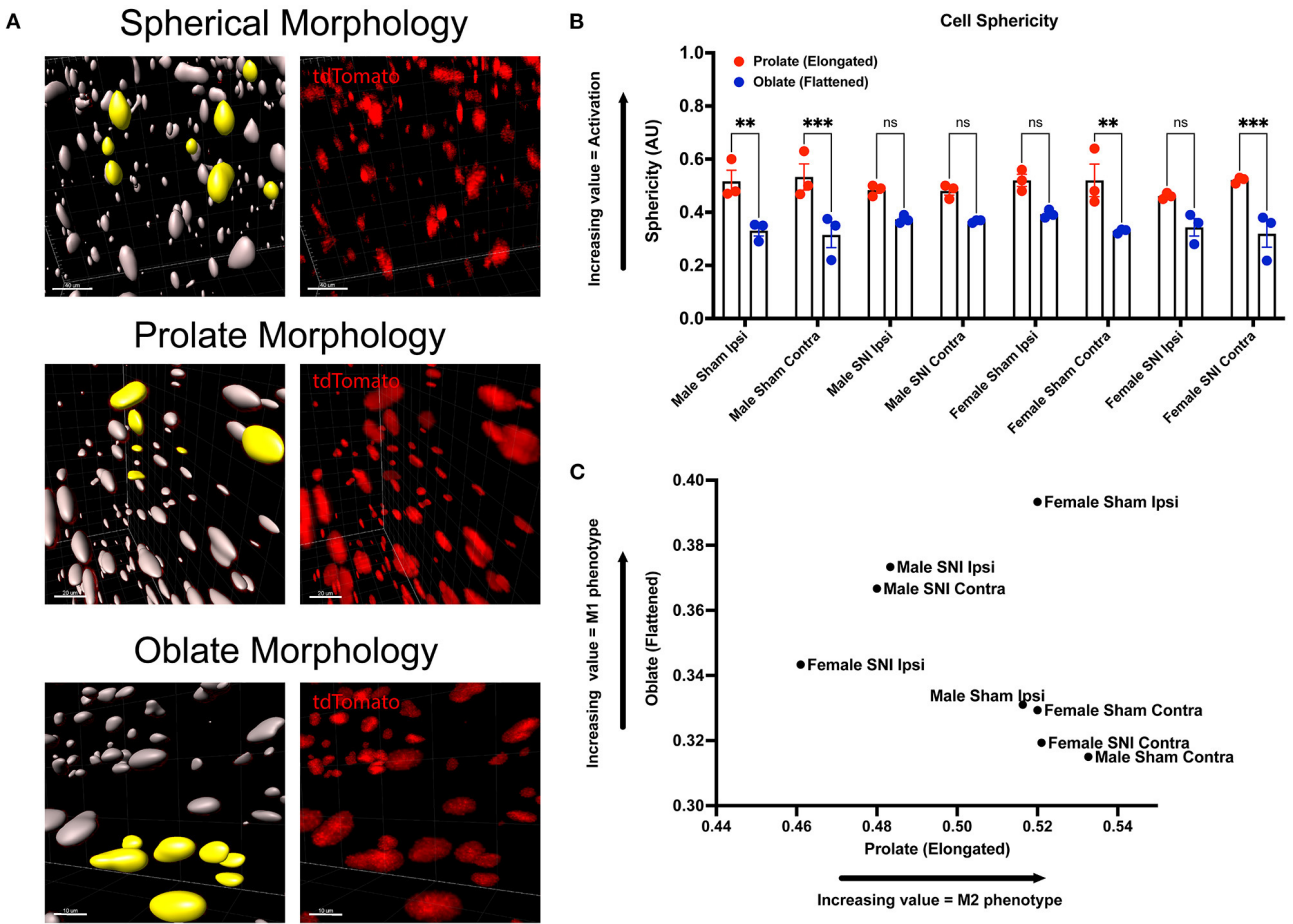
Morphology of LysM^tdT+^ macrophages in the lumbar DRGs 5 days post-SNI. Male and female LysM^tdT+^ mice had DRGs harvested 5 days post-SNI. Tissues were processed and cleared using ScaleS1 solution for 10–14 days and imaged using 2-photon microscopy. **(A)** Representative morphology from male and female LysM^tdT+^ (red; 1,100 nm) DRGs (*n* = 3). **(B)** Imaris analysis of LysM^tdT+^ cell morphology in male and female DRGs. Cells were analyzed for three morphologic parameters (sphericity, prolate, and oblate) and are displayed comparing oblateness and prolateness with respect to sphericity. **(C)** Scatter plot of DRG LysM^+^ cell oblateness with respect to prolateness in both male and female injury and control groups. ^**^p < 0.01; ^***^p < 0.001. Scale Bar: 100 μm. IP, Ipsilateral; CO, Contralateral.

Next, we wanted to identify changes in macrophage morphology after SNI as these changes can be correlated with shifts in pro-(M1) and anti-(M2) inflammatory polarization. Macrophages have been shown to play a key role in regulating homeostasis and tissue repair after nerve injury, and polarization plays an integral role in this process (Chernykh et al., 2016). We may map changes in geometric profiles of macrophages using measures of oblate (flattened) ellipticity, prolate (elongated) ellipticity, and sphericity (Iwata et al., 2017). In response to physiologic input and cytokine signaling, M2 polarized macrophages are associated with an elongated, prolate morphology while M1 macrophages are associated with an oblate, flattened morphology (Bertani et al., 2017). Drawing upon these recent discoveries and classifications, we assessed the geometrics of these recruited LysM^tdT+^ macrophages in the DRG using Imaris analysis (Figure 3A). We found differences in the clustering of LysM^tdT+^ macrophages in mice that received SNI, which take on a more oblate (flattened) morphology after nerve injury. This is indicative of an M1 polarized phenotype. Moreover, we found that LysM^tdT+^ macrophages in animals that received a sham surgery take on a more prolate (elongated) morphology. This is indicative of an M2 polarized phenotype (Figure 3C). Lastly, we assessed sphericity of LysM^tdT+^ macrophages after nerve injury (Figure 3B). Although we find no significant differences, cells that take on a more prolate (flattened) shape after injury are also more spherical, indicating a more active phenotype (Sen et al., 2016). Taken together, we conclude that macrophages have a dynamic morphological response to injury and may be characterized based on their geometric shape.

### SNI Induces Dynamic Changes in Macrophage Morphology in the ScN

**Figure 4:**
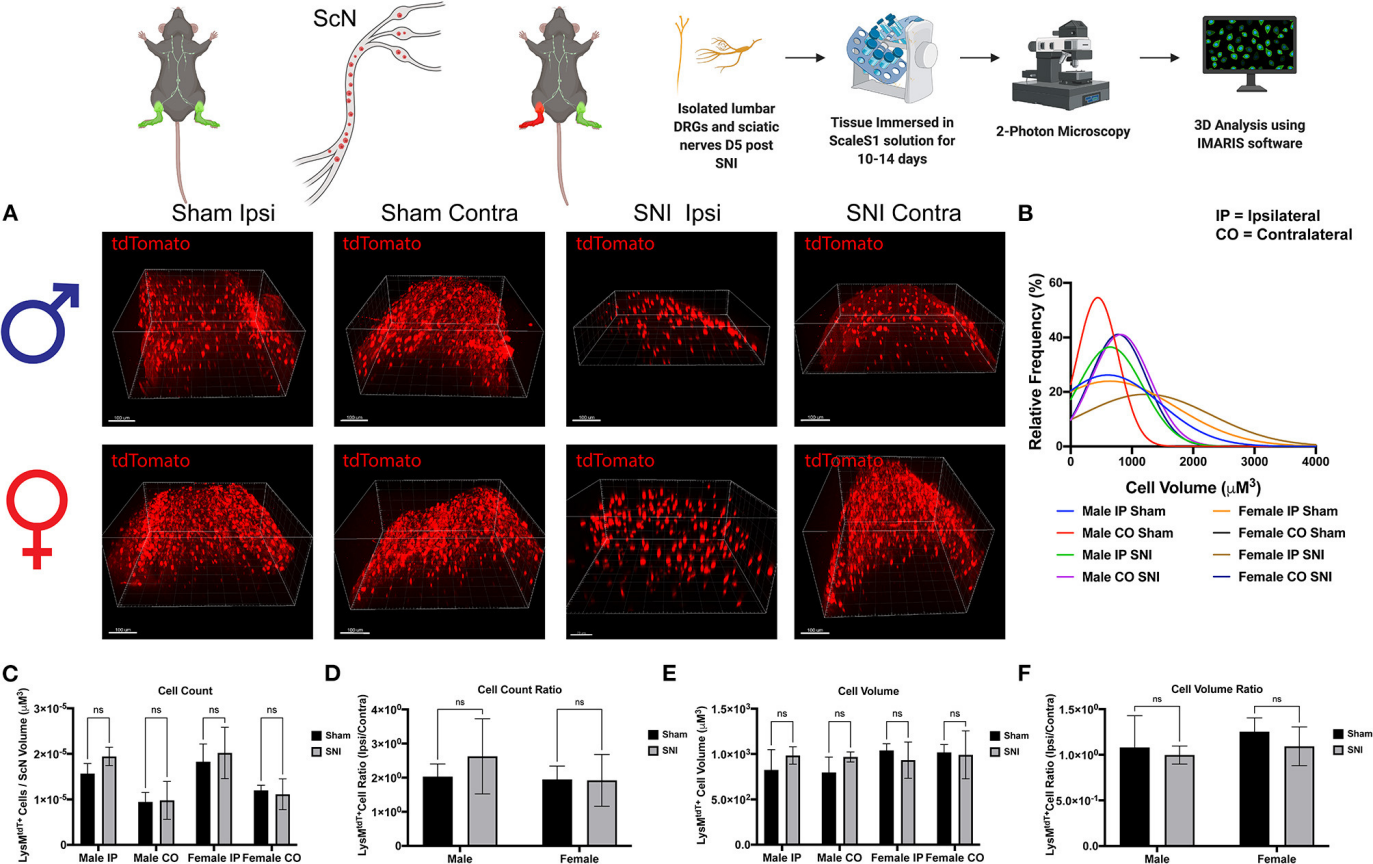
Infiltration of LysM^tdT+^ immune cells in the ScNs 5 days post-SNI. Male and female LysM-cre × tdTomato mice had ScNs harvested 5 days post-SNI. Tissues were processed and cleared using ScaleS1 solution for 10–14 days and imaged using 2-photon microscopy. **(A)** Representative images from male and female LysM^tdT+^ (red; 1,100 nm) ScNs (*n* = 3). **(B)** Frequency distribution histogram comparing the frequencies of LysM^tdT+^ cell volumes in control and injured male and female lumbar ScNs. **(C)** Quantification of LysM^+^ immune cell infiltration in the lumbar ScNs 5 days post-SNI (*n* = 3) in males and females. **(D)** Quantification of LysM^tdT+^ macrophage infiltration in the lumbar ScNs 5 days post-SNI as a ratio of ipsilateral over contralateral ScN (*n* = 3) in males and females. **(E)** Quantification of LysM^tdT+^ macrophage volume in the lumbar ScNs 5 days post-SNI (*n* = 3) in males and females. **(F)** Quantification of LysM^tdT+^ macrophage cell volumes in the ScN 5 days post-SNI as a ratio of ipsilateral over contralateral ScN (*n* = 3) in males and females. Scale Bar: 100 μm. IP, Ipsilateral; CO, Contralateral. Made using BioRender.com.

To further identify the dynamic role of macrophages in response to nerve injury, we looked closer to the site of injury in the ScN. Here, we sought to distinguish the LysM^tdT+^ macrophage response between the DRG and ScN. Using the same principles, whole ScNs were harvested from male and female LysM^tdT+^ mice 5 days after SNI and were cleared using Sca*l*eS1. Tissues were cleared and imaged using multiphoton imaging and tissues were analyzed using Imaris (Figure 4A). Analyses revealed no significant differences in LysM^tdT+^ macrophage recruitment to the distal or proximal ScN. Although, both male female mice exhibit elevated LysM^tdT+^ macrophages in the ipsilateral ScN as compared to the contralateral control. While not significant, this does indicate a surgery-induced upregulation in macrophage recruitment to the ScN in males and females (Figures 4C,D). We report no differences in the volumes of these recruited LysM^tdT+^ macrophages (Figure 4D). Moreover, we do not report any differences in the distribution or relative frequencies of LysM^tdT+^ macrophage volumes after SNI in the ScN. However, female mice exhibit a larger distribution of LysM^tdT+^ cell volumes after SNI in the ipsilateral ScN as compared to other groups, indicating increased classical activation of macrophages (Figure 4B). Lastly, we report no differences in the cell volumes of LysM^tdT+^ macrophages in the ScN after SNI in both males and females (Figures 4E,F). Taken together, we can conclude there are dynamic biological changes that occur in the ScN following SNI in males and females.

**Figure 5:**
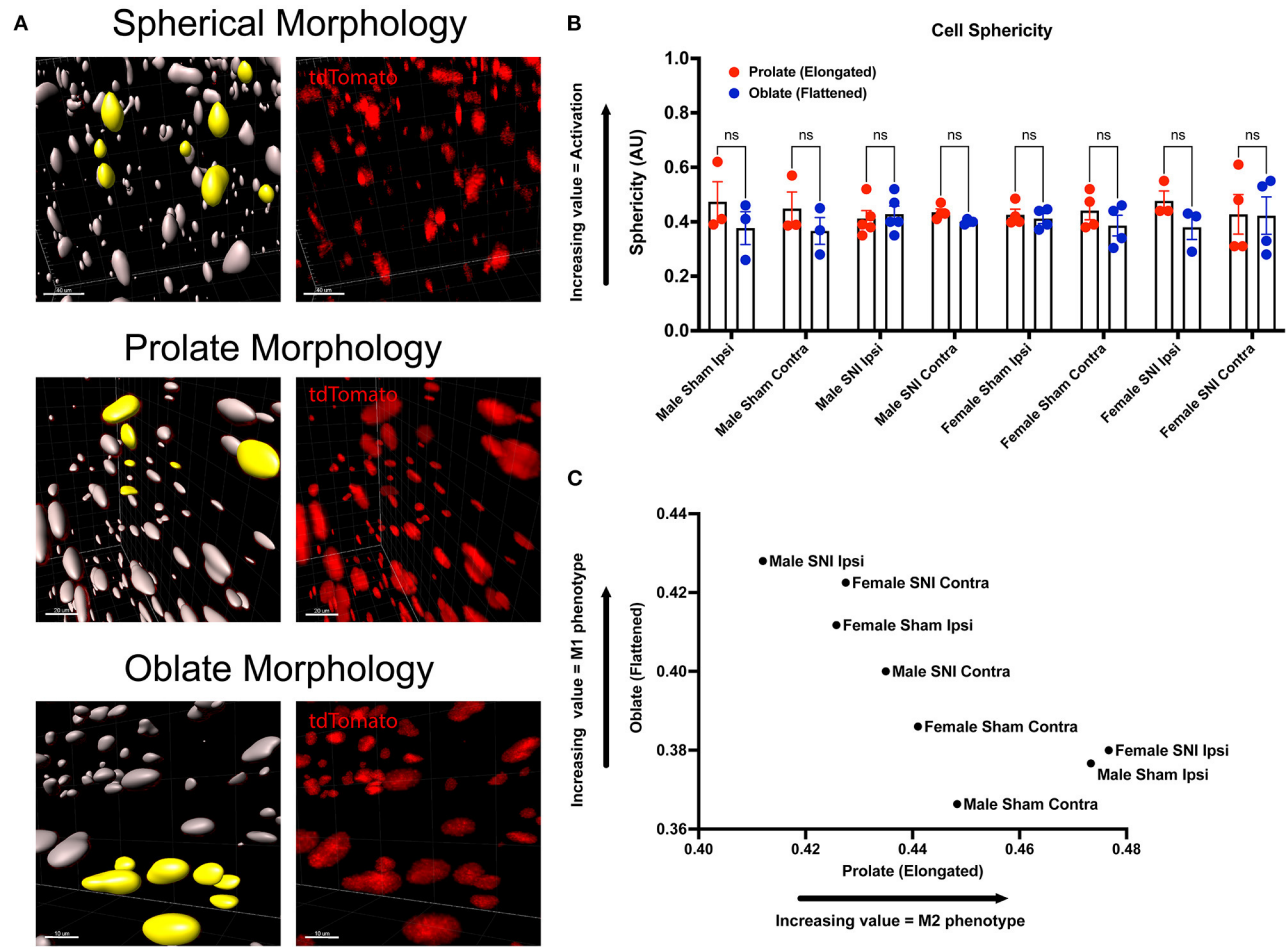
Morphology of LysM^tdT+^ macrophages in the ScN 5 days post-SNI. Male and female LysM^tdT+^ mice had ScNs harvested 5 days post-SNI. Tissues were processed and cleared using ScaleS1 solution for 10–14 days and imaged using 2-photon microscopy. **(A)** Representative morphology from male and female LysM^tdT+^ (red; 1,100 nm) ScNs (*n* = 3). **(B)** Imaris analysis of LysM^tdT+^ cell morphology in male and female ScNs. Cells were analyzed for three morphologic parameters (sphericity, prolate, and oblate) and are displayed comparing oblateness and prolateness with respect to sphericity. **(C)** Scatter plot of ScN LysM^+^ cell oblateness with respect to prolateness in both male and female injury and control groups. Scale Bar: 100 μm. IP, Ipsilateral; CO, Contralateral.

We incorporated our geometric analyses to better understand the morphological changes that may occur in macrophages in the ScN (Figure 5A). While we report no differences in recruitment, there may be morphological changes that are biologically relevant (Cobos et al., 2018). We investigated the sphericity of LysM^tdT+^ macrophages in the ScN and found that there were no differences between sexes or injury. Typically, a more spherical, or round, macrophage is classically activated, however, this does not mean that these macrophages have not altered their contractile state in response to injury. We report that there is a sexual dimorphism in the morphology of LysM^tdT+^ macrophages in the sciatic nerve 5 days after SNI. Male LysM^tdT+^ macrophages on the injured side exhibit more oblate, or flattened, cell morphology as opposed to females that exhibit a more prolate, or elongated, cell morphology (Figure 5C). This is indicative of an M1 polarization state in males as opposed to an M2 state in females. This idea has been a dogma in the field of neuroimmunology for decades (Li et al., 2009). Lastly, we find no differences in the sphericity of LysM^tdT+^ macrophages in the ScN (Figure 5B). This data indicates a distinction in macrophages in the DRG vs. ScN, where DRG macrophages are more M1 polarized in both sexes after surgery, and ScN macrophages are M1 polarized in males vs. M2 polarized in females.

## Discussion

**Figure 6:**
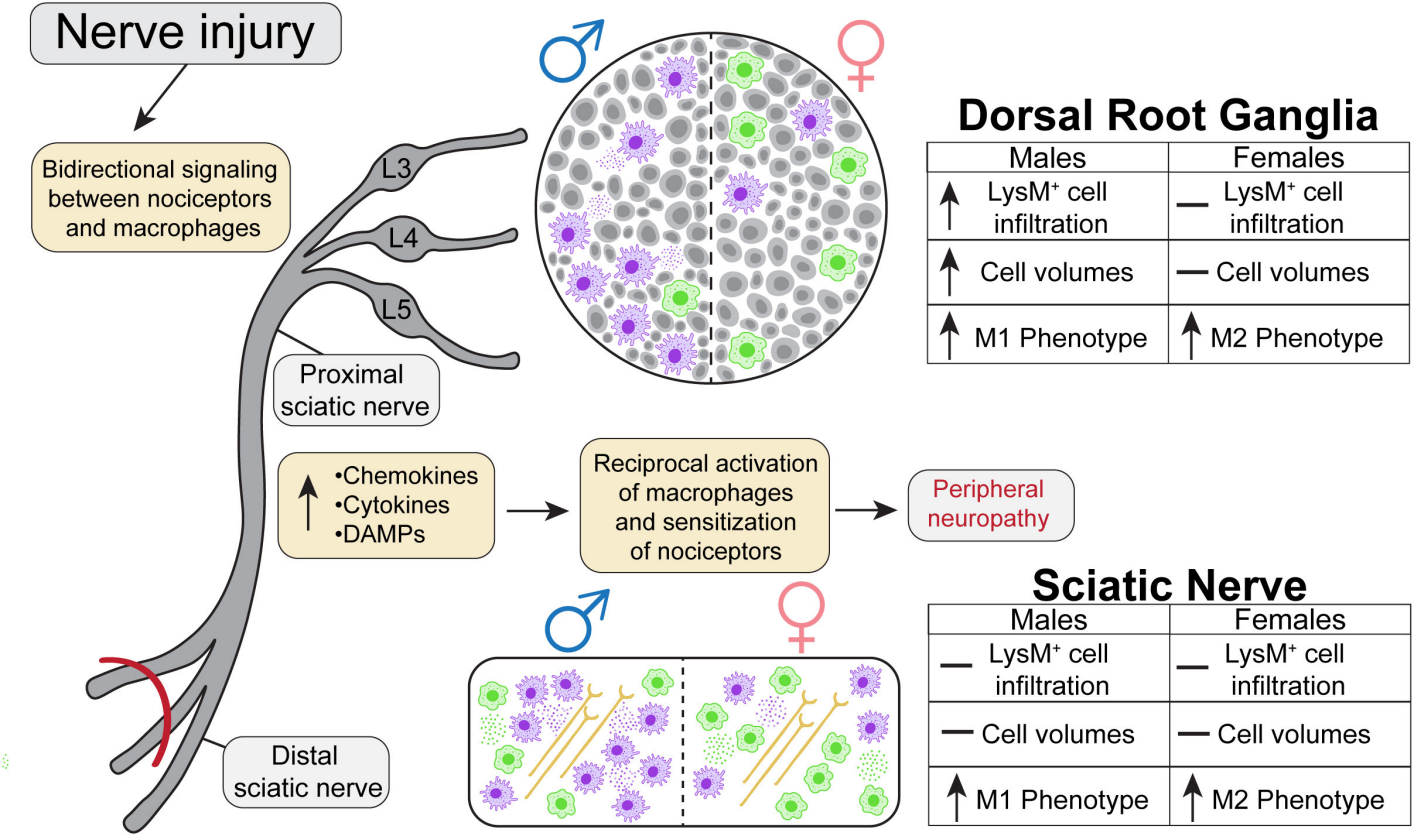
Graphical abstract representing the dynamic nature of macrophage recruitment and morphological changes following SNI in males and females.

Whole tissue *in-situ* visualization can reveal neuroimmune interactions that have been overlooked in the past. The goal of the present study was to analyze the sex-dependent dynamics of macrophage recruitment and changes in morphology after injury utilizing next generation tissue clearing and imaging techniques. We performed SNI on male and female transgenic reporter mice constitutively expressing tdTomato, a red fluorescent protein, in macrophages under the LysM promoter. We then harvested lumbar DRGs and ScNs from these animals 5 days after the surgery and optically cleared the tissues using Sca*l*eS1. Macrophage recruitment and subsequent expansion peaks between days 3 and 7 after injury, therefore we chose day 5 to assess their physical characteristics (Chen et al., 2015). We then acquired high resolution Z-stack images of the cleared tissues using multiphoton microscopy and performed extensive analyses using Imaris Imaging Software to understand the dynamics of macrophage recruitment, activation, and morphology after injury. In this study, we found that males have higher LysM^tdT+^ macrophage counts in the lumbar DRGs (L4/5), expanding upon current literature that suggests females have alternative neuroimmune mechanisms that contribute to pain states. Moreover, we find dynamic changes in LysM^tdT+^ macrophage morphology, with SNI inducing a more pro-inflammatory, or M1, phenotype measured by cell shape (Figure 6). This highlights the need to improve our understanding of the sex-dependent distribution and role of other immune cells in pain. From a therapeutic perspective, there is a potential to harness the abilities of macrophages to induce anti-nociception and tissue repair after injury (Yu et al., 2020). The vast majority of our current understanding in macrophage functionality is reliant on soluble factors, such as cytokine production and cell surface protein expression. While this information is useful in providing the necessary framework to investigate the molecular underpinnings of macrophage activation in response to injury, bridging the gap between functionality and physical characteristics will discern the full spectrum of their involvement in tissue injury.

Bi-directional communication between neuronal and non-neuronal cells in the DRG is a core mechanism in mediating the mammalian response to injury (Lopes et al., 2017). Current literature highlights the complex role that macrophages play in regulating inflammation and pro-nociception following injury, but the relevant sites of action remains unclear (Echeverry et al., 2013). Moreover, the current dogma in the field dictates males utilize a myeloid cell driven mechanism of neuronal sensitization to drive neuroinflammation (McWhorter et al., 2013). Although the reported behavioral contribution of these DRG macrophages is not dimorphic in nature, there exists a key cellular difference in their recruitment a morphology, lending to the idea that etiology differences can influence pain perception over time. Our data shows the magnitude of injury-induced macrophage expansion in the lumbar DRGs is significantly higher in males than in females. These findings are also supported by a recent paper that characterizes sex differences in immune cell recruitment to both central and peripheral nervous tissues through flow cytometric analyses (Mosser and Edwards, 2008). They demonstrate an upregulation of macrophages in both males and females after peripheral nerve injury in the lumbar DRGs, with key sex differences in the adaptive immune response. However, the adaptive immune response to injury was not the focus of our study. Collectively, these studies support the idea that macrophage recruitment to the DRG plays a critical role in neuroinflammation. Furthermore, we utilized sham and contralateral data to determine that no conclusive sex differences exist in baseline cell size and volumes further strengthening our premise that immune response to injury is sex dependent.

Conflicting reports about the role of macrophages in the ScN after nerve injury has groups reporting that macrophages do not play an important role in the pathogenesis of nerve injury-induced pain (Yu et al., 2020), while other reports found that inhibiting local macrophages in the ScN impairs development of neuropathic pain following nerve injury (Paul et al., 2013). An important distinction here is that we assessed the recruitment and morphological changes in macrophages at both distal ScN and proximal ScN. Here we assessed differences in macrophage activity with more granularity than previous studies. We find no significant recruitment of LysM^tdT+^ macrophages in the ScN 5 days after SNI, however, this does not equate to a lack of biological significance. It is a potential that the differences in macrophage morphology after SNI may alter cytokine signaling. Certain M2 macrophages phenotypes, or cells that have an elongated shape, retain the ability to produce pro-inflammatory cytokines and may be a differentiating factor in the immune response to injury in males vs. females (de Paoli et al., 2014). Despite our collective efforts, a rift exits in our understanding of the role of macrophages in the ScN, but with every advance we are able to better understand the nuances of macrophages recruitment, activation and subsequent biological implications.

While the aforementioned studies have significantly improved our understanding of the dynamic nature of macrophage recruitment to peripheral nervous tissues, they have inherent limitations that we have addressed using our model. Geometric alteration of macrophages in response to injury has the ability regulate their functional phenotype (Tauer, 2002). However, it is impossible to discern this type of information by using typical immunohistochemical or flow cytometric analyses. Macrophages exhibit an elongated shape when polarized to an M2 phenotype, as opposed to a flattened shape when polarized to an M1 phenotype (Bronte and Murray, 2015). These polarization states are involved in a myriad of biological processes related to inflammation and tissue repair and are often indicative of a macrophages change in contractility as they interact with the extracellular matrix and cell adhesion molecules (Jensen and Berg, 2017). *In vivo* these polarization states are not dichotomous, but targeted therapeutics may be better tailored to treat each sex individually. Moreover, traditional imaging acquisition and analysis techniques are not well-suited to investigate changes in cellular shape as there are significant limitations in the resolution and depth of acquired images. This severely dampens the ability of researchers to utilize common image analysis software for this purpose. Therefore, we sought to address this lack of granularity by improving our understanding of the physical characteristics of macrophages in response to nerve injury utilizing Imaris image analysis (Belle et al., 2017).

Optical clearing techniques provide additional clarity in resolving cell to cell interactions by circumventing the limitations of traditional 2-dimensional (2-D) imaging. Typically, 2-D image reconstruction of limited z-frames provides biased information as cellular density is not uniform throughout select regions of interest. By using Sca*l*eS1 optical tissue clearing in combination with deep tissue multiphoton microscopy, we were able to create a 3-D model of whole DRGs and ScNs. This allowed us to accurately assess the infiltration of immune cells to these tissues following peripheral nerve injury. Multiphoton microscopy may be used to image thicker tissue slices, but image quality quickly deteriorates when focusing deeper into a sample (Renier et al., 2014). To circumvent this limitation, we chose to utilize Sca*l*eS1 as our method of optical tissue clearing. Sca*l*eS1 was our preferred method of optical tissue clearing as it has been shown to avoid tissue expansion, preserve lipids and provide a safe immersion-medium for objectives (Hama et al., 2015). We also took into consideration other clearing methods, such as DBE or CLARITY. These alternate clearing methods offered a shorter clearing time and work better on larger tissue sizes, however they had considerable limitations with regard to preservation of fluorescence and tissue integrity (Jensen and Berg, 2017). Another clearing method we considered was DISCO which utilizes a de-lipidating agent to enhance the refractory index of the tissue (Belle et al., 2017). Similar to previously discussed alternatives, this method allows for faster clearing times, however it is accompanied with significant amounts of tissue distortion making it suboptimal for our study. Current use of this method is better suited for larger tissues, such as brain or embryos (Renier et al., 2014). Furthermore, Sca*l*eS1 utilizes materials that are inexpensive and commonly found in research labs making it easy to use and more accessible to a wider audience. Moreover, Sca*l*eS1 is well-suited for imaging fluorescent proteins, which was the primary focus of our study.

In conclusion, the combined use of our genetic model, tissue clearing, and multiphoton microscopy serves as a powerful tool for investigating neuroimmune spatiotemporal relationships, and provides a versatile framework to further our understanding of the role macrophages play in pain development. Moreover, we further delineate the sexual dimorphisms that exist in the physical phenotype of macrophages in response to nerve injury.

## Data Availability Statement

The raw data supporting the conclusions of this article will be made available by the authors, without undue reservation.

## Ethics Statement

The animal study was reviewed and approved by The University of Texas at Dallas; Institutional Animal Care and Use Committee.

## Author Contributions

TAS-P and UMS performed experiments and updated protocol equally. TAS-P, UMS, and ZWC analyzed data and wrote the manuscript. MDB oversaw all aspects of the project, conceived and originally designed the project and acquired funding. All authors contributed to the manuscript and have approved the submitted version.

## Conflict of Interest

The authors declare that the research was conducted in the absence of any commercial or financial relationships that could be construed as a potential conflict of interest.

## Supplementary Material

Supplementary Figure 1 3-Dimensional video render of a cleared Male Sham Ipsilateral DRG. Visualized cells are LysM^tdT+^ macrophages infiltrating into the tissue.

Supplementary Figure 2 3-Dimensional video render of a cleared Male Sham Contralateral DRG. Visualized cells are LysM^tdT+^ macrophages infiltrating into the tissue.

Supplementary Figure 3 3-Dimensional video render of a cleared Male SNI Ipsilateral DRG. Visualized cells are LysM^tdT+^ macrophages infiltrating into the tissue.

Supplementary Figure 4 3-Dimensional video render of a cleared Male SNI Contralateral DRG. Visualized cells are LysM^tdT+^ macrophages infiltrating into the tissue.

Supplementary Figure 5 3-Dimensional video render of a cleared Female Sham Ipsilateral DRG. Visualized cells are LysM^tdT+^ macrophages infiltrating into the tissue.

Supplementary Figure 6 3-Dimensional video render of a cleared Female Sham Contralateral DRG. Visualized cells are LysM^tdT+^ macrophages infiltrating into the tissue.

Supplementary Figure 7 3-Dimensional video render of a cleared Female SNI Ipsilateral DRG. Visualized cells are LysM^tdT+^ macrophages infiltrating into the tissue.

Supplementary Figure 8 3-Dimensional video render of a cleared Female SNI Contralateral DRG. Visualized cells are LysM^tdT+^ macrophages infiltrating into the tissue.

## Abbreviations

SNI: spared-nerve injury
DRG: dorsal root ganglia
ScN: sciatic nerve
IL: interleukin
LysM: LysozymeM
PFA: paraformaldehyde
PBS: phosphate-buffered saline
IP: ipsilateral
CO: contralateral.
